# Supplementary material for: Bio-Functionalized Chitosan for Bone Tissue Engineering
Source: Int J Mol Sci. 2021 May 31;22(11):5916. doi: 10.3390/ijms22115916 (PMC8198664; doi:10.3390/ijms22115916)
Supplement: Supplementary file 1 [file ijms-22-05916-s001.zip › ijms-1220205-supplementary.pdf]

Supporting Materials

Biofunctionalized Chitosan for Bone Tissue Engineering

Paola Brun [1, *], Annj Zamuner [2], Chiara Battocchio [3], Leonardo Cassari [2], Martina Todesco [2], Valerio Graziani [3], Giovanna Iucci [3], Martina Marsotto [3], Luca Tortora [3], Valeria Secchi [3] and Monica Dettin [2]

[1] Department of Molecular Science, University of Padua, Via A. Gabelli 63, 35121 Padua, Italy
[2] Department of Industrial Engineering, University of Padua, Via F. Marzolo 9, 35131 Padua, Italy
[3] Department of Science, Roma Tre University of Rome, Via della Vasca Navale 79, 00146 Rome, Italy
* Correspondence: paola.brun.1@unipd.it; Tel.: +39-0498272343

Table S1. XPS results.

Sample	Signal	Peak	BE (eV)	FWHM (eV)	Atomic ratios		
					A/A_{max}	A/At_{ot}	A/σ
**RGD**	C1s	1	285.00	1.60	7.27	0.61	618250
		2	286.48	1.60	2.97	0.25	252647
		3	287.95	1.60	1	0.08	85080
		4	289.29	1.60	0.64	0.05	54218
	O1s	1	530.02	1.72	0.66	0.18	25454
		2	532.08	1.72	0.71	0.47	65880
		3	533.50	1.72	1	0.28	38511
		4	535.01	1.72	0.25	0.07	9501
	N1s	1	399.17	2.04	0.13	0.08	2701
		2	400.36	2.04	1	0.63	20981
		3	402.80	2.04	0.45	0.28	9401
**Chit-RGD**	C1s	1	285.00	1.66	0.12	0.02	144735
		2	286.70	1.66	4.59	0.74	5550539
		3	288.40	1.66	1	0.16	1208321
		4	290.32	1.66	0.48	0.08	585063
	O1s	1	530.82	1.94	0.15	0.07	134399
		2	533.26	1.94	0.89	0.42	770565
		3	534.50	1.94	1	0.48	868436
		4	536.33	1.94	0.05	0.02	40962
	N1s	1	399.87	1.66	0.06	0.05	9384
		2	401.47	1.66	1	0.76	157082
		3	402.58	1.66	0.24	0.19	38224
**HVP**	C1s	1	285.00	1.37	20.17	0.82	923238
		2	286.48	1.37	2.60	0.11	119123
		3	288.00	1.37	1	0.04	45783
		4	289.12	1.37	0.78	0.03	35720
	O1s	1	529.98	1.51	0.06	0.05	5470
		2	531.00	1.51	0.08	0.06	6453
		3	532.44	1.51	1	0.78	85281
		4	533.92	1.51	0.16	0.12	13661
	N1s	1	398.93	1.53	0.18	0.14	4090
		2	400.26	1.53	1	0.80	23333
		3	401.71	1.53	0.43	0.06	1757
**Chit-HVP**	C1s	1	285.00	1.68	3.17	0.45	3734125
		2	286.29	1.68	2.80	0.39	3299717
		3	288.13	1.68	1	0.14	1176988
		4	289.54	1.68	0.12	0.02	146517
	O1s	1	529.69	1.82	0.04	0.02	59605
		2	531.64	1.82	0.61	0.35	1008717
		3	532.88	1.82	1	0.59	1665187
		4	534.39	1.82	0.07	0.04	108780
	N1s	1	398.62	1.88	0.09	0.08	101703
		2	400.06	1.88	1	0.88	1176933
		3	402.08	1.88	0.05	0.05	60571

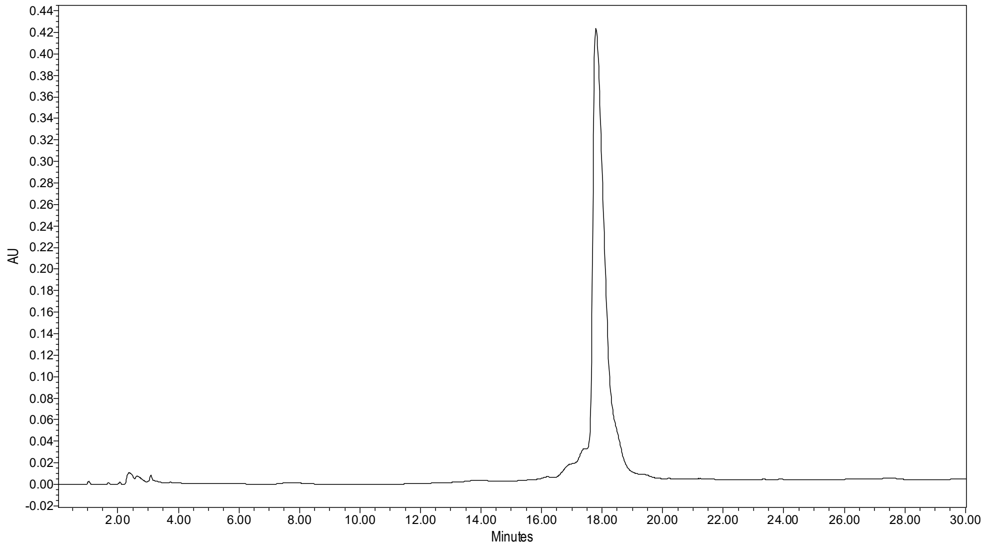

Figure S1. Analytical RP-HPLC chromatogram of purified GRGDSPK-aldehyde peptide. The analysis conditions were: NovaPak C_{18} Column (Waters, Milford, Massachusetts, USA), flow of 1 mL/min, eluent A composed of 0.05% TFA in H_2O milliQ, eluent B composed of 0.05% TFA in CH_3CN, injection of 100 µl of GRGDSPK-aldehyde dissolved in milliQ water (1 mg/mL), gradient from 0% to 15% of eluent B in 30 minutes, absorbance acquired at 214 nm.

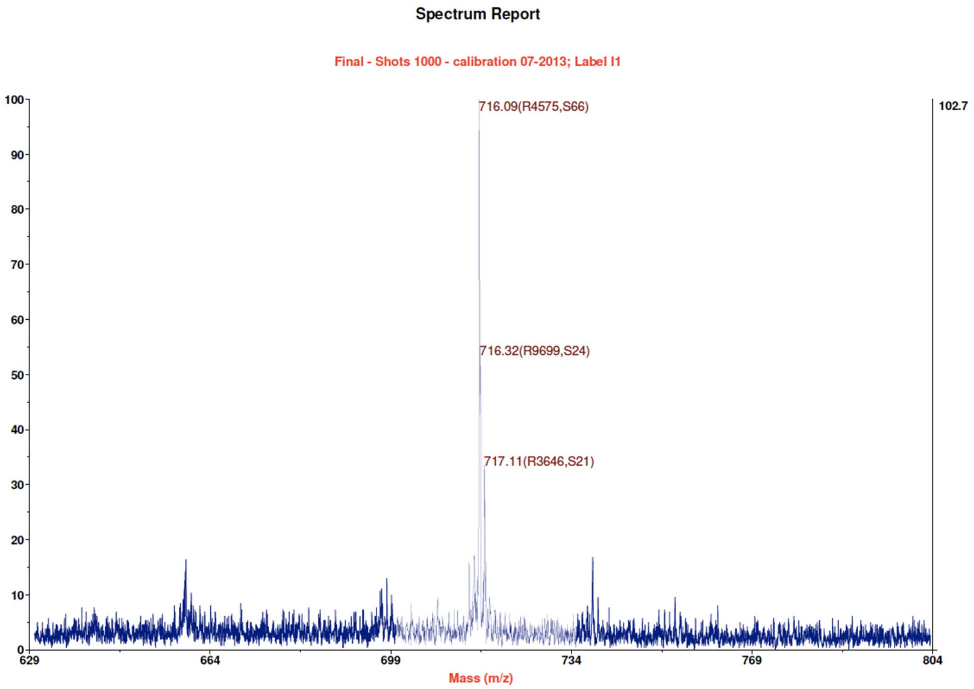

Figure S2. Mass analysis of GRGDSPK-aldehyde peptide. MALDI-TOF evaluation confirmed the identity of the purified peptide. GRGDSPK-aldehyde peptide: experimental mass = 716.09 Da, theoretical mass = 714.77 Da.

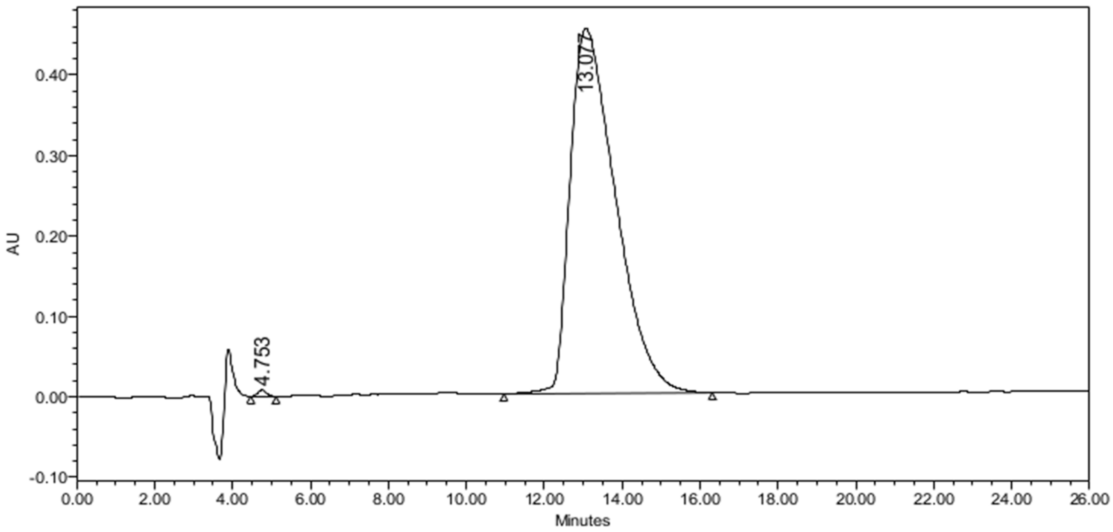

Figure S3. Analytical RP-HPLC chromatogram of purified HVP-aldehyde peptide. The analysis conditions were: Jupiter C_{18} Column (Phenomenex, Torrance, CA 90501-1430, USA), flow of 1 mL/min, eluent A composed of 0.05% TFA in H_2O milliQ, eluent B composed of 0.05% TFA in CH_3CN, injection of 100 µl of HVP-aldehyde dissolved in milliQ water (1 mg/mL), gradient from 15% to 28% of eluent B in 26 minutes, absorbance acquired at 214 nm.

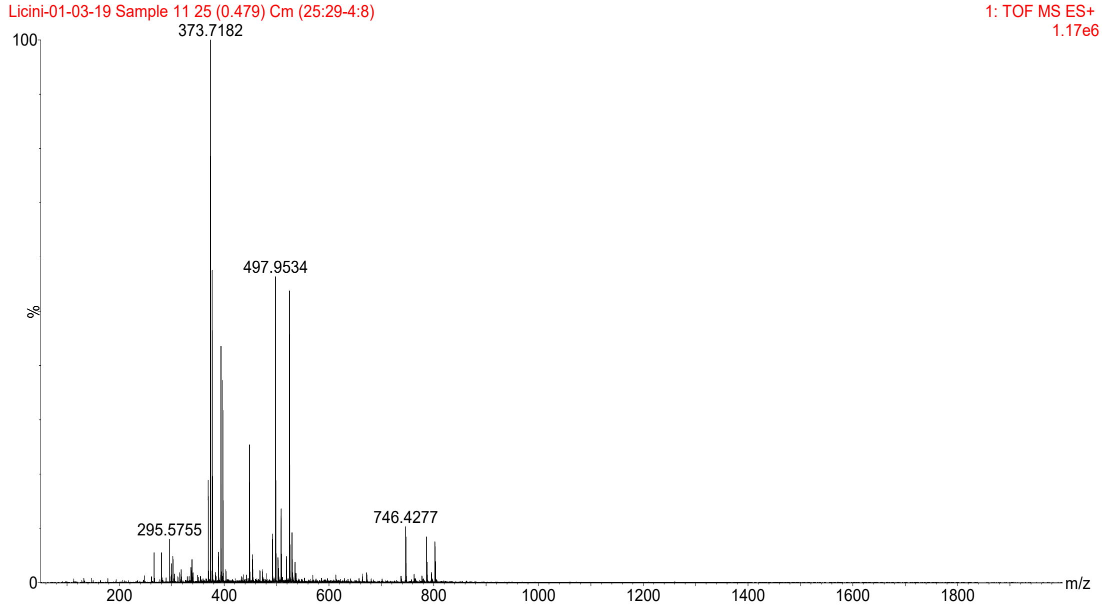

Figure S4. Mass analysis of HVP-aldehyde peptide. ESI-ToF mass spectrum reporting m/z peaks relating to HVP-aldehyde peptide. HVP-aldehyde peptide: experimental mass = 1491.85 Da, theoretical mass = 1491.78 Da.
